# Effect of Spp1 on nerve degeneration and regeneration after rat sciatic nerve injury

**DOI:** 10.1186/s12868-017-0348-1

**Published:** 2017-03-07

**Authors:** Xingyu Liu, Yuhua Sun, Huaiqin Li, Yuting Li, Meiyuan Li, Ying Yuan, Shusen Cui, Dengbing Yao

**Affiliations:** 1 China-Japan Union Hospital of Jilin University, 126 Xiantai Road, Changchun, 130033 Jilin People’s Republic of China; 2 School of Life Sciences, Jiangsu Key Laboratory of Neuroregeneration, Co-Innovation Center of Neuroregeneration, Nantong University, 19 Qixiu Road, Nnatong, 226001 Jiangsu People’s Republic of China; 3 Affiliated Hospital of Nantong University, 20 Xisi Road, Nantong, 226001 Jiangsu People’s Republic of China

**Keywords:** Wallerian degeneration, Secreted phosphoprotein 1 (Spp1), Schwann cells, Nerve regeneration, Rat, Sciatic nerve injury

## Abstract

**Background:**

Wallerian degeneration (WD) in injured peripheral nerves is associated with a large number of up- or down-regulated genes, but the effects of these changes are poorly understood. In our previous studies, we reported some key factors that are differentially expressed to activate nerve degeneration and regeneration during WD. Here, we determined the effects of secreted phosphoprotein 1 (Spp1) on WD after rat sciatic nerve injury.

**Results:**

Spp1 was upregulated from 6 h to 14 days after sciatic nerve injury. Altered expression of Spp1 in Schwann cells (SC) resulted in altered mRNA and protein expression levels for cytokines, c-Fos, PKCα and phospho-ERK/ERK and affected SC apoptosis in vitro. Silencing of Spp1 expression in SCs using siRNA technology reduced proliferation and promoted migration of SCs in vitro. By contrast, overexpression of Spp1 promoted proliferation and reduced migration in SCs in vitro. Differential expression of Spp1 after sciatic nerve injury in vivo altered the expression of cytokines, c-Fos, PKCα, and the p-ERK/ERK pathway.

**Conclusions:**

Spp1 is a key regulatory factor that affects nerve degeneration and regeneration through c-Fos, PKCα and p-ERK/ERK pathways after rat sciatic nerve injury. These results shed new light on the role of Spp1 in nerve degeneration and regeneration during WD.

## Background

Wallerian degeneration (WD), the degeneration of the axon distal to a site of transaction, occurs in both axons and myelin after injury to the peripheral nervous system (PNS) [1–3]. The PNS, unlike the central nervous system (CNS), is capable of regeneration after an injury that causes WD processes to begin [4, 5]. A number of studies have found that nerve injury not only plays a key role in modulating the activities of Schwann cells but also promotes axonal regeneration by releasing a large number of regeneration-related factors, including cytokines, growth factors, and chemokines [5–7]. Therefore, it is important to elucidate the key factors involved in regulating the degeneration and regeneration that occurs in the PNS after injury [8–11]. The molecular mechanisms regulating WD are not yet completely understood, but understanding the factors that regulate rapid responses during WD may reveal the mechanisms underpinning nerve repair and regeneration [12–16].

Secreted phosphoprotein 1 (Spp1) belongs to the family of secreted acidic proteins. It has a large number of consensus sequence sites and multiple phosphorylation sites and binds to several integrin receptors, which have been well established to function in cell adhesion, migration, and survival [17]. Spp1 is expressed in a range of immune cells and reported to act as an immune modulator, which promotes cell recruitment to inflammatory sites [17]. It also functions as an adhesion protein involved in cell attachment and wound healing [17, 18]. Stimulation of Spp1 expression leads to an increase in cell pro-inflammatory cytokine levels, although the regulatory pathways are not yet known [17–19].

We previously reported on gene expression signal flow and pathways regulated by key factors as determined by microarray analyses, such as claudins, transforming growth factor beta 1, Spp1, and toll-like receptor 4, during the processes of WD after rat sciatic nerve injury [11–15]. Here, we examined the effect of Spp1 on cytokine release, cell apoptosis, cell migration and proliferation, and signaling pathways in vitro and in vivo.

## Methods

### Animal model

Male Sprague–Dawley rats (180–200 g) were provided by the Experimental Animal Center of Nantong University. The rats were randomly divided into eight groups (six rats per group) and underwent sciatic neurectomy. All animal tests were conducted in accordance with the US National Institutes of Health’s *Guide for the Care and Use of Laboratory Animals* and by the *Key Laboratory of Neuroregeneration Guidelines for the Care and Use of Laboratory Animals*. The Institutional Animal Care and Use Committee of Nantong University approved all protocols used in this study.

The rats were anesthetized using an injection of complex narcotics (85 mg/kg trichloroacetaldehyde monohydrate, 42 mg/kg magnesium sulfate, and 17 mg/kg sodium pentobarbital), and the sciatic nerve was identified and lifted through an incision on the lateral aspect of the mid-thigh of the right hind limb. The sciatic nerve was cut, and a 1-cm segment was excised. One group of rats was immediately used in experiments (0 h), and the other groups were used 6, 12, and 24 h as well as 1, 2, 3, and 4 weeks after the surgery. The 0-h animals received sham operations.

### Primary culture of Schwann cells

The rats for this experiment were provided by the Experimental Animal Center of Nantong University. The rats were sacrificed, and Schwann cells (SCs) were isolated from the sciatic nerves. The SCs were treated with anti-Thy1.1 antibody (Sigma, St Louis, MO) and rabbit complement (Invitrogen, Carlsbad, CA) to remove fibroblasts as previously described [20]. SCs were cultured from the sciatic nerves of 1-day-old Sprague–Dawley rats as previously described [20]. Primary cultures of Schwann cells were maintained in Dulbecco’s modified Eagle’s medium (DMEM) supplemented with 10% fetal bovine serum at 37 °C in a humidified 5% CO_2_ atmosphere.

### Spp1 siRNA transfection of Schwann cells

Three different small interfering RNAs (siRNAs) (Table 1) were used to perform RNA interference. SCs were transfected with Spp1 siRNAs (Integrated Biotech Solutions, Shanghai, China) using Lipofectamine RNAi MAX transfection reagent (Invitrogen, Carlsbad, CA, USA) according to the manufacturer’s instructions. Black control that raised normally and NC-siRNA were tested. The siRNA-1, 2, 3 transfection experiments were repeated three times.

**Table 1:** Spp1 siRNA primers

Gene	Sequence
NC	F: 5′ UUCUCCGAACGUGUCACGUTT 3′ R: 5′ ACGUGACACGUUCGGAGAATT 3′
Spp1-1	F: 5′ CUAGAUGUCGGUGUCCCUUGC 3′ R: 5′ UGAAUGUUGCUGCGCAUCAUG 3′
Spp1-2	F: 5′ CGAUCGAUAGUGCCGAGAAGC 3′ R: 5′ UUCUCGGCACUAUCGAUCGCA 3′
Spp1-3	F: 5′ AGCUAGUCCUAGACCCUAAGA 3′ R: 5′ UUAGGGUCUAGGACUAGCUUG 3′

### Overexpression of Spp1 in Schwann cells

SCs were cultured in DMEM (GIBCO, Grand Island, NY) with 100 IU/mL penicillin, 10% fetal calf serum, and 100 g/mL streptomycin at 37 °C and 5% CO_2_. The SCs were identified by examining the immunofluorescence of an antibody to the marker S100, and the final cells were found to comprise 98% SCs. The Spp1 overexpression plasmid pcDNA3.1-Spp1 was constructed as previously described [21]. A mixture of pcDNA3.1-Spp1 plasmid and X-treme GENE HP DNA Transfection Reagent (Roche, Mannheim, Germany), or X-treme GENE HP DNA Transfection Reagent and an empty vector were then transfected into SCs for 48 h. After that, real-time quantitative (q)PCR and Western blot analyses were conducted. The pcDNA3.1-Spp1 overexpression experiments were repeated three times.

### Real-time quantitative PCR analysis

Total RNA was extracted with Trizol regent, and cDNA was synthesized with a cDNA Reverse Transcription Kit (Qiagen, Valencia, CA, USA). Real-time qPCR was performed using a 7300 Real-Time PCR System according to the manufacturer’s protocols. The analysis was repeated three times, and the reactions were conducted in triplicate. The comparative Ct method was used to analyze the cycle threshold (Ct) values. The data were analyzed, and group differences were considered statistically significant at values of *p* less than 0.05.

### Western blot analysis

Injured nerve samples and SCs were homogenized in protein lysis buffer containing protease inhibitors. The protein expression levels were analyzed using antibodies against anti-Spp1, AKT, phosphorylated (p)-AKT, protein kinase C-alpha (PKCα), c-Fos, extracellular signal-regulated kinase (ERK), and p-ERK. The Western blot images were scanned with a GS800 Densitometer Scanner (Bio-Rad, Hercules, CA, USA), and the optical density data were analyzed using PDQuest 7.2.0 software. GAPDH was used as a reference to normalize the levels of protein. The data were analyzed, and group differences were considered statistically significant at values of *p* less than 0.05. All injured nerve samples were analyzed in three independent experiments.

### Flow cytometry analysis

The extent of SC apoptosis was measured using an Annexin V-FITC Apoptosis detection kit (Beyotime Institute of Biotechnology, China) as described by the manufacturer’s instructions. SCs were washed with PBS and then collected for flow cytometry analysis. FITC-labeled annexin V (5 μL) in binding buffer (195 μL) was incubated for 10 min at room temperature. The incubation was continued with 10 μL of propidium iodide for 10 min on ice in the dark. After that, the apoptotic cells were measured by FACScan flow cytometry.

### Cell proliferation assay

Cultured SCs were plated at a density of 2 × 10^5^ cells/mL onto 0.01% poly-l-lysine-coated plates. Cell proliferation was assayed at 2 days after cell transfection. EdU (50 µM) was added to the cell culture and incubated for 2 h. The SCs were then fixed with 4% formaldehyde for 30 min. After SC labeling, a Cell-Light EdU DNA Cell Proliferation Kit (Ribobio, China) was used to analyze cell proliferation according to the manufacturer’s protocol. Cell proliferation was expressed as the ratio of EdU-positive cells, which was defined by images of randomly selected fields obtained on a DMR fluorescence microscope (Leica Microsystems, Bensheim, Germany). The cell proliferation assays were performed three times using triplicate wells.

### Cell migration assay

Transwell chambers (6.5 mm) with 8-µm pores were used to examine SC migration as described previously [21]. SCs (10^6^ cells/mL) resuspended in 100 µL of DMEM were transferred to the top chamber and allowed to migrate in 5% CO_2_ into the lower chamber before the addition of 600 µL complete medium. Cells adhering to the bottom surface of each membrane were stained with 0.1% crystal violet, imaged, and counted using a DMR inverted microscope (Leica Microsystems, Bensheim, Germany). The cell migration assays were conducted three times using triplicate wells.

### Immunohistochemistry

The distal sciatic nerve samples were fixed with 4% paraformaldehyde and dehydrated in 30% sucrose solution. Sections were cut using a cryostat to a thickness of 12 µm and mounted onto slides. The sections were rinsed in PBS, permeabilized in 0.3% Triton X-100, 5% goat serum, and 1% BSA in PBS, and then stained. The sections were incubated with mouse monoclonal anti-S100 (1:400, Sigma) and Spp1 (1:50, Santa Cruz) antibodies at 4 °C for 12 h, and then incubated with goat anti-mouse or goat anti-rabbit IgG Cy3 (1:400, Sigma) and IgG Alexa Fluor 488 (1:400, Invitrogen) at room temperature for 2 h. The sections were counterstained with Hoechst 33342 for 5 min. All samples were observed under a fluorescence microscope. Images were acquired using a laser microscope (FV10i-oil, Tokyo, Japan).

### In vivo assay

The sciatic nerve of adult male Sprague–Dawley rats was exposed through an incision on the left hind limb and cut to create a 1-cm gap. A silicone tube (i.d., 1.0 mm) was implanted to bridge the nerve gap. The rats were randomly divided into two groups (n = 3 each): Spp1 siRNA injected into the tube after the nerve gap bridge for the experimental group, and a control group. At 7 and 14 days after surgery, the rats were killed, and the silicone tubes together with the regenerated nerves were collected. Real-time PCR and Western blot analyses were conducted. The nerve samples (7 and 14 days) were analyzed in three independent experiments.

### Statistical analysis

Statistical analyses were performed using SPSS 15.0 for windows (SPSS, Chicago, IL, USA). Group differences were analyzed by one-way analysis of variance and Scheffé’s post hoc test when appropriate. Student’s *t* test was used for comparisons between two groups. Values of *p* less than 0.05 were considered statistically significant. All data are expressed as mean ± SD.

## Results

### Spp1 is expressed in injured sciatic nerves and in Schwann cells

**Fig. 1:**
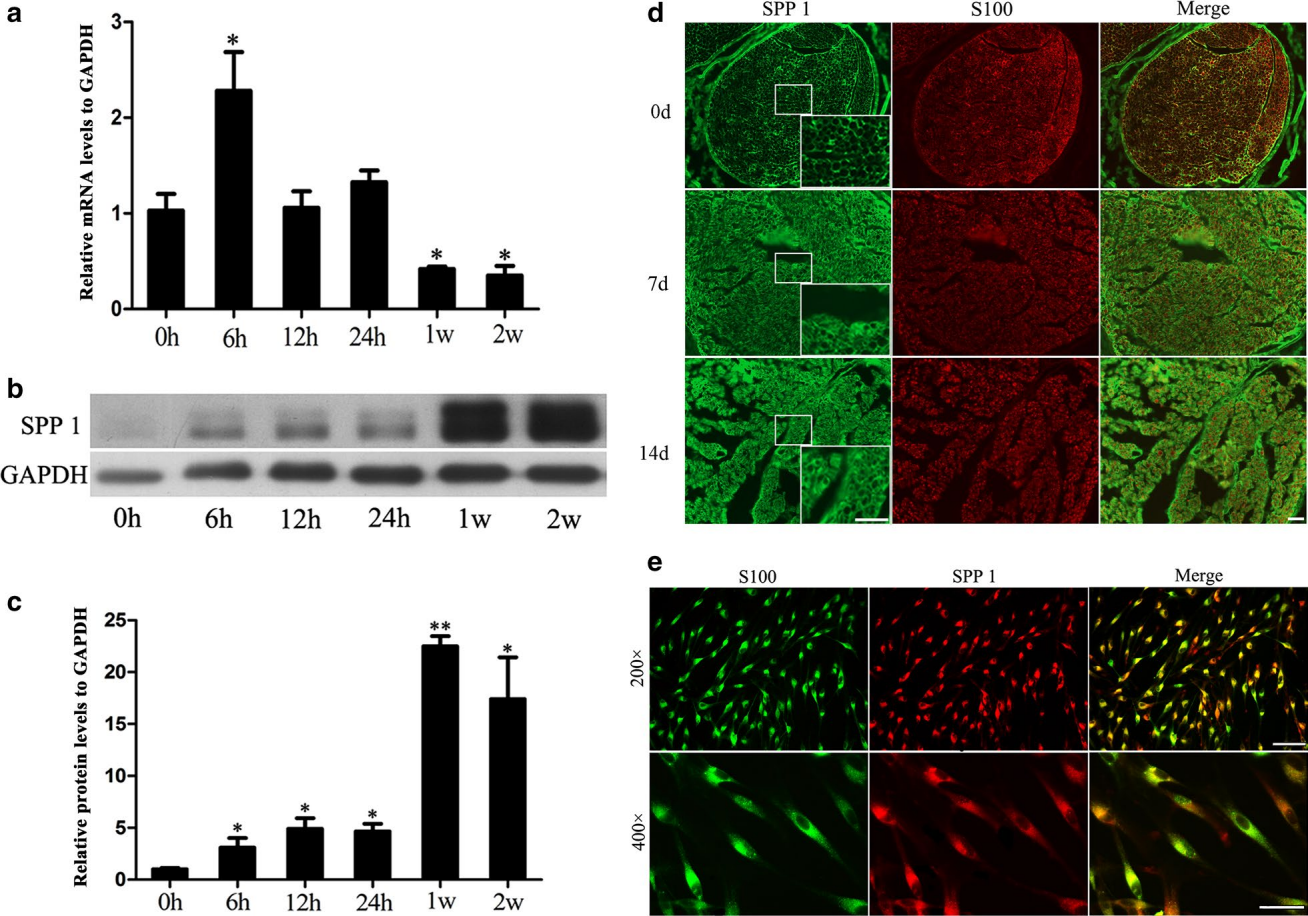
Spp1 expression in injured rat sciatic nerve and cultured Schwann cells. **a** Real-time qPCR analysis of Spp1 expression in injured sciatic nerves 0, 6, 12, and 24 h as well as 1 and 2 weeks post injury. GAPDH was used to normalize the data. The average of three independent experiments is shown as mean ± SEM (*p < 0.05). **b** Western blot analysis of Spp1 expression levels in injured sciatic nerves 0, 6, 12, and 24 h as well as 1 and 2 weeks post injury. GAPDH levels were used as a control. **c** Relative protein expression levels of Spp1 in Western blot analyses. **d** Immunofluorescence staining of S100 and Spp1 (and their overlay) in distal sciatic nerve stumps of rats at the indicated times (14 and 28 days) and in the normal sciatic nerve. S100 was used as an SC-specific marker. Spp1 is colocalized in the plasma membrane of S100-positive Schwann cells (*scale bar* 50 μm). **e** Immunofluorescence staining of S100 (*green*) and Spp1 (*red*) (and their overlay) in cultured Schwann cells (*scale bar* 50 μm). Each experiment was repeated three times

We used real-time qPCR and Western blot analyses to determine the expression of Spp1 0, 6, 12, and 24 h as well as 1 and 2 weeks after sciatic nerve injury. The real-time qPRC results indicated that Spp1 mRNA expression was increased from 6 to 24 h after injury and then decreased. Spp1 mRNA level was significantly higher at 6 h after injury. Our western blot assay results indicated that Spp1 protein expression was increased from 6 h to 2 weeks. Spp1 protein level was significant higher at 1 w after injury. GAPDH levels were used as a control (Fig. 1a–c). We used immunohistochemistry to visualize the location of Spp1 and S100 at 0, 14, 28 days after sciatic nerve injury and in cultured Schwann cells. The Schwann cells were immunostained with anti-S100, which is a specific Schwann cell marker. The results of our immunostaining assay demonstrated that Spp1 and S100 were colocalized in Schwann cells (Fig. 1d), indicating that Spp1 is expressed in the Schwann cells of the sciatic nerve. We also examined the expression of Spp1 in cultured Schwann cells. The data indicated that Spp1 was also present in cultured Schwann cells (*p* < 0.05, vs. day 0; Fig. 1e). All data were analyzed using one-way analysis of variance and Scheffé’s post hoc tests (*p < 0.05).

### Spp1 knockdown and overexpression in transfected SCs alters mRNA expression levels

**Fig. 2:**
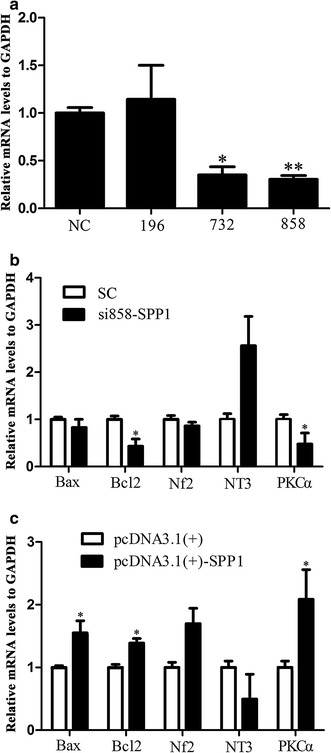
Cytokine expression in SC Spp1 knockdown and overexpression. **a** Spp1 siRNA-1, 2, 3 transfection efficiency assay (*p < 0.05). **b** Real-time qPCR analysis of *bax*, *bcl2*, *nf2*, *NT3*, and *PKCα* mRNA expression levels after Spp1 siRNA-858 transfection of SCs for 2 days. GAPDH was used to normalize values to the negative control (*p < 0.05). The average of three independent experiments is shown as mean ± SEM. **c** Real-time qPCR analysis of *bax*, *bcl2*, *nf2*, *NT3*, and *PKCα* mRNA expression levels after pcDNA3.1-Spp1 plasmid transfection of SCs for 2 days, using GAPDH to normalize data to the negative control (*p < 0.05). The average of three independent experiments is shown as mean ± SEM

We synthesized three specific Spp1 siRNAs—siRNA-1, siRNA-2 and siRNA-3—and all three were found to reduce Spp1 mRNA expression levels. The interference transfection efficiencies of two of the siRNA fragments were at least 80% (Fig. 2a); thus, we selected the most efficient one, siRNA-3 (siRNA-858), for the following experiment. To investigate the potential functions of Spp1 in SCs, including on cytokine release, we analyzed the mRNA levels of the pro-apoptotic factors B-cell lymphoma 2 (Bcl2) and Bcl-2-associated X protein (Bax) as well as neurofibromin 2 (Nf2), neurotrophin 3 (NT3) and PKCα after Spp1 knockdown and overexpression in transfected SCs. Our real-time qPCR results showed that *bax*, *bcl2*, *Nf2* and *PKCα* mRNA expression levels were downregulated in SCs with Spp1 knocked down and upregulated by Spp1 overexpression in SCs. By contrast, the expression of *NT3* mRNA was upregulated by Spp1 knockdown and downregulated by Spp1 overexpression in SCs (Fig. 2b, c) (*p < 0.05). These data indicated that the differential expression of Spp1 alters mRNA expression levels in SCs.

### Spp1 affects c-Fos, PKCα, and ERK signaling pathways in vitro

**Fig. 3:**
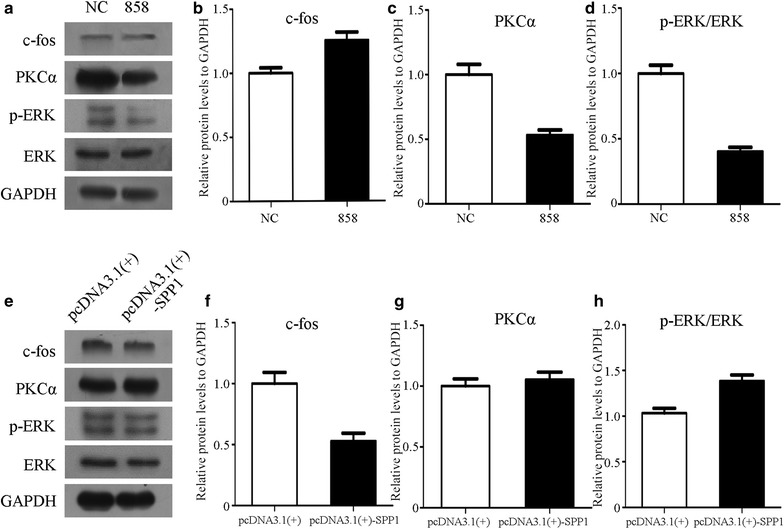
Expression of c-Fos, PKCα and p-ERK/ERK protein levels in SC Spp1 knockdown and overexpression. **a** Western blot analysis of c-Fos, PKCα and p-ERK/ERK expression after Spp1 siRNA-858 transfection of SCs for 2 days, using GAPDH to normalize data to negative controls. The average of three independent experiments is shown as mean ± SEM. **b**–**d** Relative protein expression levels of c-Fos, PKCα, and p-ERK/ERK as determined by Western blot analysis. **e** Western blot analysis of c-Fos, PKCα, and p-ERK/ERK expression after pcDNA3.1-Spp1 plasmid transfection of SCs for 2 days, using GAPDH to normalize data to negative controls. The average of three independent experiments is shown as mean ± SEM. **f**–**h** Relative protein expression levels of c-Fos, PKCα, and p-ERK/ERK as determined by Western blot analysis

As shown above, altered Spp1 expression resulted in changes in mRNA expression levels that could affect cytokines. We next examined whether Spp1 affected signaling pathways in vitro using cultured SCs. Protein expression levels of c-Fos, PKCα and p-ERK/ERK were compared with those in negative control SCs using Western blot analysis. We found that protein levels of c-Fos, PKCα and p-ERK/ERK were significantly changed after transfection of Spp1 siRNA and the pcDNA3.1-Spp1 plasmid (*p < 0.05), indicating that c-Fos, PKCα and p-ERK/ERK signaling pathways could be activated by Spp1 (Fig. 3). Thus, Spp1 may play roles in regulating c-Fos, PKCα, and p-ERK/ERK signaling pathways in cultured SCs in vitro.

### Spp1 affects SC apoptosis, proliferation, and migration in vitro

**Fig. 4:**
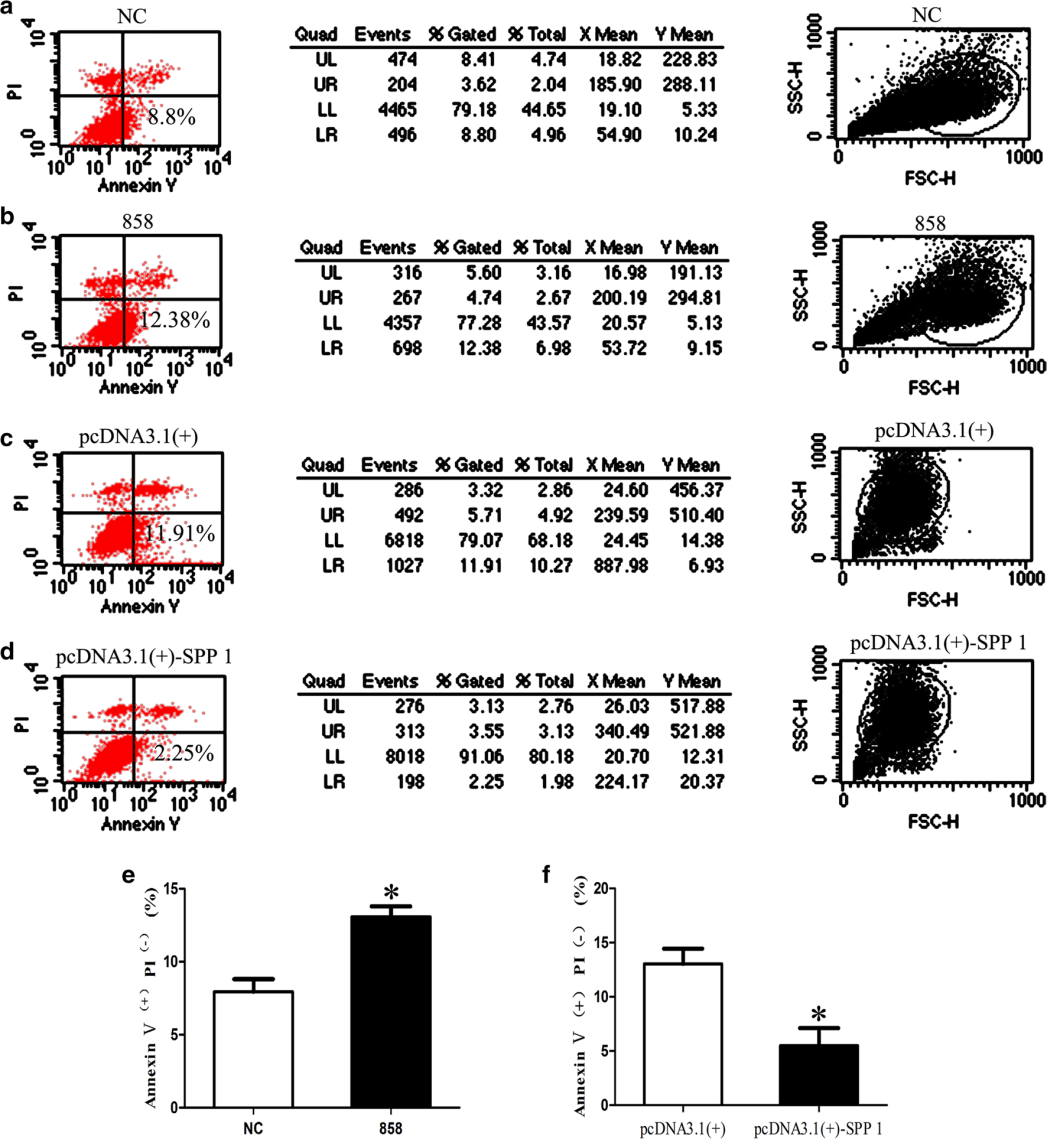
Spp1 knockdown and overexpression affect SC apoptosis. **a**, **b** Silencing Spp1 by transfection with Spp1 siRNA significantly induces apoptosis of SCs compared with that in negative controls (NC). **c**, **d** Overexpression of Spp1 in SCs by transfection with the pcDNA3.1-Spp1 plasmid significantly inhibits SC apoptosis compared with that in NCs. **e** Relative numbers of SCs with the Spp1 knockdown. **f** Relative numbers of SCs overexpressing Spp1. The average of three independent experiments is shown as mean ± SEM (*p < 0.05)

**Fig. 5:**
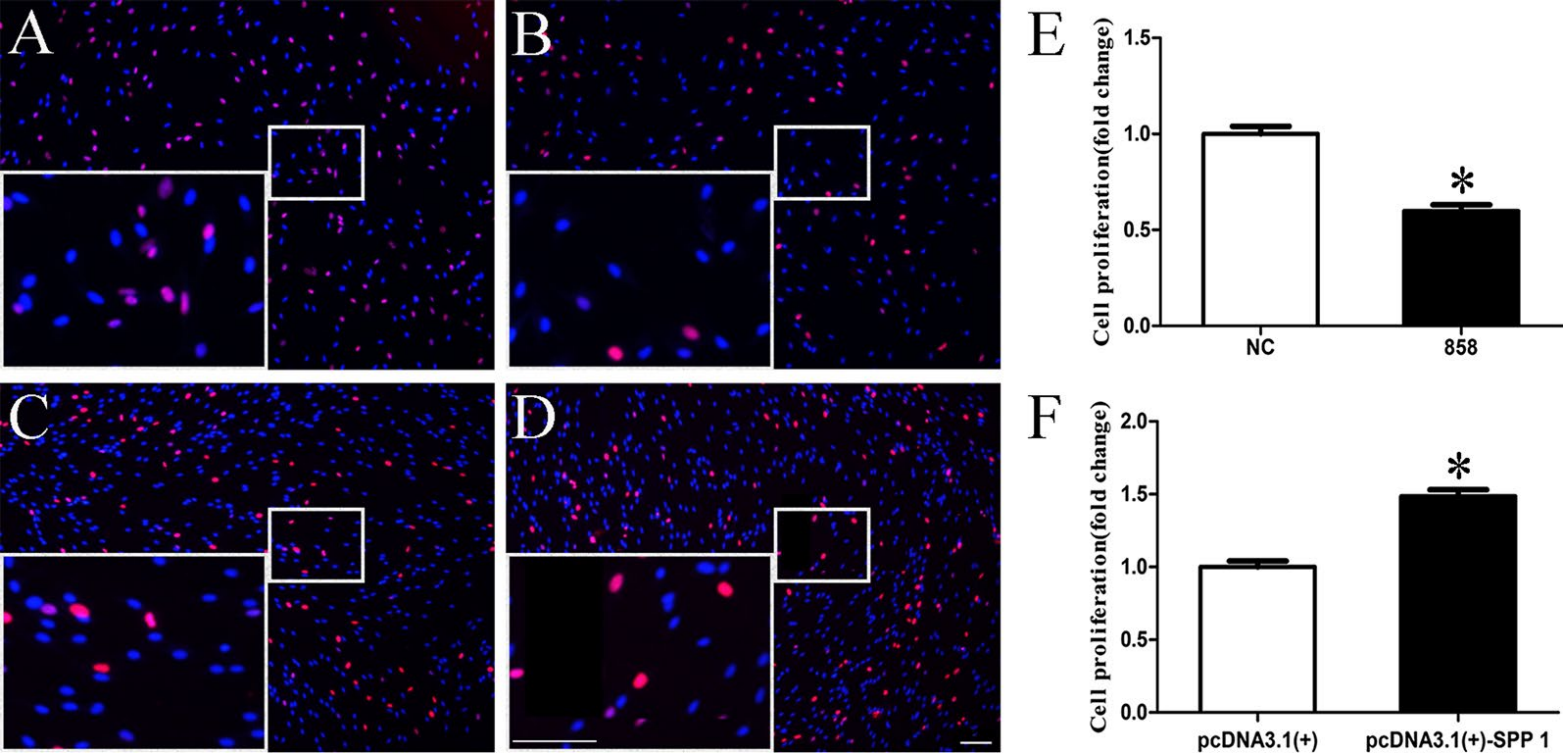
Spp1 knockdown and overexpression affect SCs proliferation. **a**, **b** Silencing of Spp1 by transfection with Spp1 siRNA significantly inhibits SC proliferation compared with that in negative controls (NCs). **c**, **d** Overexpression of Spp1 by transfection with the pcDNA3.1-Spp1 plasmid significantly induces SC proliferation compared with that in NCs. **e** Relative numbers of SCs with the Spp1 knockdown. **f** Relative numbers of SCs overexpressing Spp1 (*scale bar* 100 μm). The average of three independent experiments is shown as mean ± SEM (*p < 0.05)

**Fig. 6:**
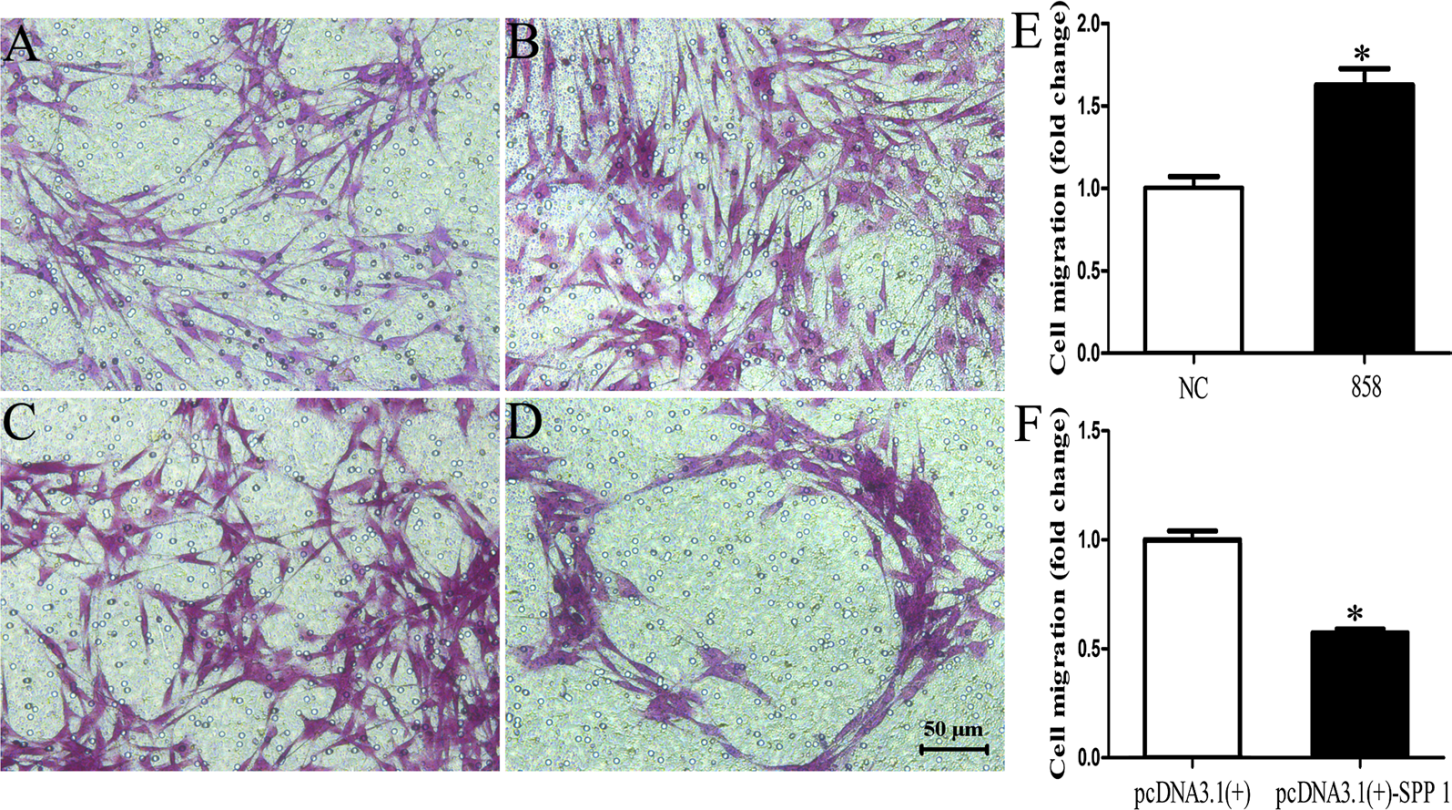
Spp1 knockdown and overexpression affect SCs migration. **a**, **b** Silencing of Spp1 by transfection with Spp1 siRNA in SCs significantly induces SC migration compared with that in negative controls (NCs). **c**, **d** Overexpression of Spp1 by transfection with the pcDNA3.1-Spp1 plasmid significantly inhibits SC migration compared with that in NCs. **e** Relative numbers of SCs with the Spp1 knockdown. **f** Relative numbers of SCs overexpressing Spp1. The average of three independent experiments is shown as mean ± SEM (*p < 0.05)

To determine the function of Spp1 in SCs, primary SCs were transfected with Spp1 siRNA, pcDNA3.1-Spp1 plasmid, or negative control vector, and the effects of these transfections on cell apoptosis, proliferation, and migration of SCs were examined in vitro. Compared with that in negative controls, the apoptosis rate of SCs transfected with Spp1 siRNA was increased (1.5-fold) and that of SCs transfected with pcDNA3.1-Spp1 was decreased (2.0-fold; Fig. 4) (*p < 0.05), suggesting that silencing Spp1 induced SC apoptosis, whereas enhancing Spp1 expression reduced SC apoptosis. The results of our EdU-based proliferation assay indicated that compared with the proliferation rate of SCs in negative controls, that of SCs transfected with Spp1 siRNA was decreased (1.8-fold), whereas that of SCs transfected with the pcDNA3.1-Spp1 plasmid was increased (1.5-fold; Fig. 5) (*p < 0.05). We also found that the migration rate of SCs transfected with Spp1 siRNA was increased (1.6-fold), whereas that of SCs transfected with the pcDNA3.1-Spp1 plasmid was decreased (1.8-fold) as compared with that of the negative control (Fig. 6) (*p < 0.05). These results indicated that the expression of Spp1 affects SC apoptosis, proliferation, and migration in vitro.

### Altered Spp1 expression affects sciatic nerve injury was been assed in vivo

**Fig. 7:**
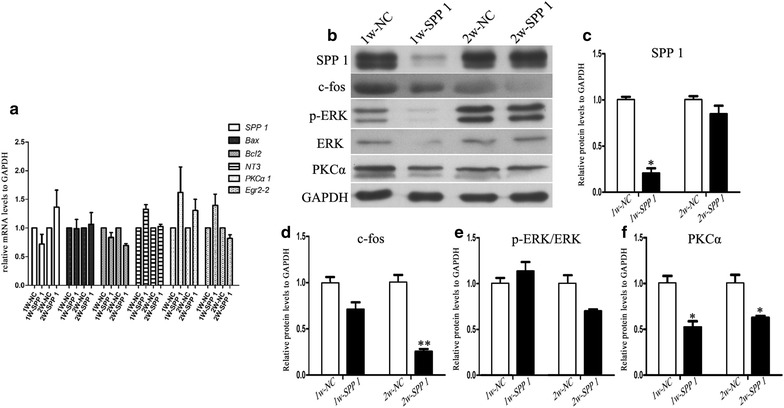
Altered Spp1 expression affects cytokine expression and c-Fos, PKCα and p-ERK/ERK pathways in vivo. **a** Real-time qPCR analysis of *Spp1*, *bax*, *bcl2*, *NT3*, *PKCα* and *EGR2* expression levels after exposure of injured rat sciatic nerves to Spp1 siRNA-858 for 1 and 2 weeks. GAPDH levels were used as a control (*p < 0.05). The average of three independent experiments is shown as mean ± SEM. **b** Western blot analysis of Spp1, c-Fos, p-ERK/ERK, and PKCα protein expression levels after injured rat sciatic nerves were exposed to Spp1 siRNA-858 for 1 and 2 weeks. GAPDH levels were used as a control (*p < 0.05). **c**–**f** Relative protein expression levels of Spp1, c-Fos, p-ERK/ERK, and PKCα as determined by Western blot analysis. The average of three independent experiments is shown as mean ± SEM (*p < 0.05)

To determine the effect of Spp1 on WD after rat sciatic nerve injury in vivo, we examined the functions of Spp1 on rat sciatic nerve repair and regeneration 1 and 2 weeks after injury. After exposing the injured sciatic nerves to either Spp1 siRNA or the negative control vector for 1 or 2 weeks, we conducted real-time qPCR and Western blot analyses. Our results indicated that altered Spp1 expression affected the mRNA and protein expression levels of the cytokines Bax, Bcl2, NT3, and early growth response 2 (EGR2) as well as of PKCα and differentially regulated the c-Fos, PKCα and p-ERK/ERK signaling pathways (Fig. 7) (*p < 0.05). These results were consistent with the functions of Spp1 observed in vitro.

## Discussion

Peripheral nerve injury and repair is a result of reactivated regeneration mechanisms in combination with newly activated injury-dependent reactions. Our data indicated that gene and protein expression changes after sciatic nerve injury appeared to provide separate signals that were characterized by a high degree of overlapping genes [22–25]. These signals are thought to recruit neutrophils and may amplify proinflammatory cytokine responses via the phosphatidylinositol 3-kinase/nuclear factor-kappa B pathway to activate the processes of nerve injury, repair, and regeneration [21, 26–33].

In the present study, we determined the effect of Spp1 on nerve repair and rejuvenation after sciatic nerve injury in vitro and vivo. Spp1 has been described as a component of the inflammatory environment of dystrophic and injured tissues [17]. Different cell types may differ in their regulatory mechanisms of the Spp1 gene. Although Spp1 is also important for the migration of neutrophils in vitro [34–42], regulation of the Spp1 gene is incompletely understood. Our results indicated that Spp1 mediates cell activation and cytokine production after sciatic nerve injury. In addition, Spp1 may act as an important anti-apoptotic factor and may prevent non-programmed cell death in inflammatory colitis. Spp1 was previously shown to act as a macrophage chemotactic factor and play an important role in mast cell migration [17]. Here, we reported the functions of Spp1 in the injured sciatic nerve during WD. Stimulation of Spp1 expression resulted in cytokine expression changes and may regulate c-Fos, PKCα and p-ERK/ERK pathways in vitro. Altered Spp1 expression was also shown to affect SC proliferation, migration, and apoptosis. We also verified these data in vivo. Further studies will be necessary to identify the key regulatory factors, how they regulate signaling pathways in vivo, and their functions during WD after peripheral nerve injury.

## Conclusions

Spp1 is differentially expressed during WD after rat sciatic nerve injury. In vitro and vivo analyses revealed that Spp1 is a key regulatory factor that affects nerve degeneration and regeneration through c-Fos, PKCα, and p-ERK/ERK pathways after rat sciatic nerve injury. We concluded that Spp1 plays important roles in peripheral nerve injury, repair, and regeneration.

## Abbreviations

Bax: Bcl-associated X protein
bcl-2: B-cell lymphoma-2
bFGF: basic fiberoblast growth factor
CNS: central nervous system
ERK: extracelluler regulated protein kinase
GAPDH: glyceraldehyde-3-phosphate dehydrogenase
JaK: Janus kinase
JNK: c-Jun NH2-terminal kinase
NF2: neurofibromin 2
NT3: neurotrophin 3
PKC: protein kinase C
PNS: peripheral nervous system
SC: Schwann cells
siRNA: small interfering RNA
Spp1: secreted phosphoprotein 1
TLR: toll like receptor
